# Is cancer a severe delayed hypersensitivity reaction and histamine a blueprint?

**DOI:** 10.1186/s40169-016-0108-3

**Published:** 2016-08-23

**Authors:** Mahin Khatami

**Affiliations:** National Cancer Institute (NCI), the National Institutes of Health (NIH), Bethesda, MD USA

**Keywords:** Aging, Allergy, Angiogenesis, Branched amino acids, Bioenergetics, Cancer, Fetal growth, Histamine, Hypersensitivity, Immune-privileged and immune-responsive tissues, Mitochondria, Placenta, Taurine, Yin and Yang of inflammation

## Abstract

Longevity and accumulation of multiple context-dependent signaling pathways of long-standing inflammation (antigen-load or oxidative stress) are the results of decreased/altered regulation of immunity and loss of control switch mechanisms that we defined as Yin and Yang of acute inflammation or immune surveillance. Chronic inflammation is initiated by immune disruptors-induced progressive changes in physiology and function of susceptible host tissues that lead to increased immune suppression and multistep disease processes including carcinogenesis. The interrelated multiple hypotheses that are presented for the first time in this article are extension of author’s earlier series of ‘accidental’ discoveries on the role of inflammation in developmental stages of immune dysfunction toward tumorigenesis and angiogenesis. Detailed analyses of data on chronic diseases suggest that nearly all age-associated illnesses, generally categorized as ‘mild’ (e.g., increased allergies), ‘moderate’ (e.g., hypertension, colitis, gastritis, pancreatitis, emphysema) or ‘severe’ (e.g., accelerated neurodegenerative and autoimmune diseases or site-specific cancers and metastasis) are variations of hypersensitivity responses of tissues that are manifested as different diseases in immune-responsive or immune-privileged tissues. Continuous release/presence of low level histamine (subclinical) in circulation could contribute to sustained oxidative stress and induction of ‘mild’ or ‘moderate’ or ‘severe’ (immune tsunami) immune disorders in susceptible tissues. Site-specific cancers are proposed to be ‘severe’ (irreversible) forms of cumulative delayed hypersensitivity responses that would induce immunological chaos in favor of tissue growth in target tissues. Shared or special features of growth from fetus development into adulthood and aging processes and carcinogenesis are briefly compared with regard to energy requirements of highly complex function of Yin and Yang. Features of Yang (growth-promoting) arm of acute inflammation during fetus and cancer growth will be compared for consuming low energy from glycolysis (Warburg effect). Growth of fetus and cancer cells under hypoxic conditions and impaired mitochondrial energy requirements of tissues including metabolism of essential branched amino acids (e.g., val, leu, isoleu) will be compared for proposing a working model for future systematic research on cancer biology, prevention and therapy. Presentation of a working model provides insightful clues into bioenergetics that are required for fetus growth (absence of external threat and lack of high energy-demands of Yin events and parasite-like survival in host), normal growth in adulthood (balance in Yin and Yang processes) or disease processes and carcinogenesis (loss of balance in Yin–Yang). Future studies require focusing on dynamics and promotion of natural/inherent balance between Yin (tumoricidal) and Yang (tumorigenic) of effective immunity that develop after birth. Lawless growth of cancerous cells and loss of cell contact inhibition could partially be due to impaired mitochondria (mitophagy) that influence metabolism of branched chain amino acids for biosynthesis of structural proteins. The author invites interested scientists with diverse expertise to provide comments, confirm, dispute and question and/or expand and collaborate on many components of the proposed working model with the goal to better understand cancer biology for future designs of cost-effective research and clinical trials and prevention of cancer. Initial events during oxidative stress-induced damages to DNA/RNA repair mechanisms and inappropriate expression of inflammatory mediators are potentially correctable, preventable or druggable, if future studies were to focus on systematic understanding of early altered immune response dynamics toward multistep chronic diseases and carcinogenesis.

## Background

There is now little dispute that aging process is associated with the combined diminutions (retarded levels or loss of effectiveness) of important hormones and metabolites, enzymes and/or receptor molecules and immune responses in organs/tissues. These changes that would lead to minor or major functional rearrangements in organ systems are known as the biological and immunological senescence. The age-associated intrinsic biological rearrangements in tissues often adversely and additionally are influenced by the accumulation of a wide variety of other internal or external stimuli, including infective, chemical, biological or environmental agents; defective proteins or genes, senescent or cancerous cells that increase the risks of induction of a wide range of chronic inflammatory conditions (e.g., neurodegenerative or autoimmune diseases, hypertension, diabetes and cardiovascular complications, stroke) or site-specific cancers.

The good news is that the increased rate of healthy older individuals around the globe suggests that the aging process is not necessarily associated with illnesses. Healthy older people can lead active and productive lives well beyond the current standard age of retirement of 65–70 years [1–3].

If the scientific and medical community took a closer look at a century-old reductionist and chaotic approaches to biomedical research and therapy for chronic diseases, particularly cancer, they would come to the same conclusion that the status quo on such fragmentary research cannot be acceptable any longer. The current failure rates of claimed ‘targeted’ therapy, ‘personalized’ or ‘precision’ medicine for solid tumors of 90 % (±5) are the outcomes of such reductionist approaches [4–6]. There is an urgent need to understand this serious problem that resulted in the loss of millions of precious lives and huge economic burden to the society [6]. Policy makers should seriously consider switching the current culture of sick care to a culture of healthcare for the aging population particularly in USA. Approaches to a healthier society are only possible if the policy makers and public understand and consider correcting the following wrong principles that have been routinely applied in medical sciences (reviewed in [6]):

1. Drastic reduction in the *might* of medical/cancer establishment that has increasingly overwhelmed the *right* of science and creation of a sick society that is drug-dependent for huge profits in the last century;
2. Systematic and integrated understanding of the details of fascinating complex biological laws that govern the body’s health provided through effective immunity for prevention or delayed onsets of age-associated diseases such as cancer and other disabling conditions throughout life.

There are enormous amount of quality isolated data on a wide range of biological topics that remain disconnected, invalidated or unused when prevention or treatment options of diseases are decided. Constructive analyses of data require understanding of the multidisciplinary biomedical fields and integration of information, and connecting the dots for developing effective roadmaps on biological pathways that control and maintain health. Such constructive efforts toward solving cancer mystery have not been possible by the reductionist and chaotic approaches to research that the current powerful cancer establishment chose to direct for a century [6]. Furthermore, producing fragmented data and feeding the information to popularized informatics and various databases by employing computational or system biology methods and expecting to find answers have not been successful and created more confusion. Despite all such efforts aging processes and development of diseases such as neurological disorders or cancers and how to control them remain mysteries to be solved [4–6].^1^ For example, except for our ‘accidental’ discoveries that were established in 1980s on experimental models of acute and chronic ocular allergies, there is little/no evidence on a direct link between inflammation and altered dynamics of immune system that would lead to cell growth and tumor [4–9]. In these studies the role of immune disruptors (antigens) on time course kinetics of developmental phases of inflammation-induced immune dysfunction resulted in tumorigenesis and angiogenesis. Recent analyses of the original data became the first report on sequential interactions and synergies between host activated immune and non-immune cells (e.g., MCs, B cells, epithelial, mucus secreting goblet cells) and those recruiting cells from vasculature (e.g., eosinophils and tumor-associated macrophages) in the direction of multistep tumorigenesis and angiogenesis [5].

Among the most scientific damaging factors that influenced lack of progress in cancer research or how to control it are the peculiar lack of interest of decision makers to systematically study the early events on initiation of altered immune response dynamics, that we defined as the loss of balance in Yin and Yang of acute inflammation, in aging process and multistep diseases, including the lawless growth of cancerous cells in susceptible tissues and development of site-specific cancers [4–9]. It is alarmingly disappointing that despite existence of over 20 million publications on cancer-related topics, and the investment of trillions of dollars for research and therapy, cancer scientists and oncologists still debate whether inflammation is protective or destructive to the body. The ongoing misunderstanding and misinformation regarding the role of inflammation has been tremendously costly as millions of lives are lost to cancer because of wrong approaches to cancer therapy that include recent reductionist approaches to immunotherapy that repeatedly failed patients [6]. The author believes that pretending and claiming that we are winning the war on cancer is worse than admitting that we are not.

The author’s recent efforts in better understanding the cancer biology include analyses and integration of valuable scattered data as well as, identification of numerous important biological gaps, toward initiation of a roadmap that would allow further probes in proposing future research directions [4–6]. Extension of such efforts is reflected in the present multi-component hypotheses that are summarized in a proposed working model to study the cancer biology from different biological angles that open new directions for future studies. In this article, we reiterate the recent proposal that nearly all age-associated chronic diseases including site-specific cancers that are manifested as distinct or different diseases are interrelated [4–6]. Unresolved or chronic inflammation (oxidative stress) will be demonstrated as a common denominator in the induction of all ‘mild’, ‘moderate’ or ‘severe’ forms of immune disorders in aging. A working model on the functionality of immune surveillance at different stages of life will be proposed with emphasis on the differential bioenergetic profiles of Yin and Yang processes and the role that mitochondria play in maintenance of health or initiation of diseases such as cancer. Circulating histamine will be considered as the blue print in the genesis of all ‘mild’, ‘moderate’ or ‘severe’ immune disorders including cancers.

## Connecting dots on age-associated diseases and cancer biology

As the organism ages, the changes in the availability, transport or quality of hormones, metabolites, nutrients, their receptor molecules and immune cell response dynamics in tissues demand biological readjustments in organ systems (biological senescence or immunosenescence). In mammals, age-associated increased in health conditions such as allergies, asthma, emphysema, hypertension, colitis, gastritis, autoimmune and neurodegenerative diseases, diabetes and cardiovascular complications, stroke, atherosclerosis, Alzheimer’s, Parkinson and site-specific cancers seem to be the manifestation of different degrees of immune disorders [4–35]. Natural aging process (immune-biological senescence) accompanies minor or major alterations in the function of immune and non-immune systems that result in altered inflammatory responses to old and new challenges in older individuals. As detailed in the following, longevity and extensive accumulation of altered crosstalk between immune and non-immune network and loss of effective immunity (immune surveillance), include impaired or loss of control in the integrities and functions of vasculature and energy power house in the mitochondria are common features of many ‘mild’, ‘moderate’ or ‘severe’ chronic health problems and site-specific cancers. We recently defined that the loss of effective immunity relates to the retardation in proper functioning of a highly complex network of hundreds of thousands of molecular signals between the biphasic response profiles of 2 biologically opposing arms, Yin (tumoricidal) and Yang (tumorigenic) properties of self terminating mechanisms of acute inflammation [6, 7]. Effective immunity is provided through innate and adaptive immune cell responses and intimately facilitated by negative and positive signals from vasculature (angiogenic and antiangiogenic), oxido-redox potentials (oxidants and anti-oxidants), as well as, metabolic and neuronal pathways [4–9]. We hypothesized that longevity and oxidative stress would lead to loss of Yin and Yang response profiles in target tissues. Chronic inflammation was suggested as a common denominator in the induction of nearly all age-associated chronic diseases or site-specific cancers [4–9]. However, how the intrinsic and extrinsic biological heterogeneities in organ systems and collective responses of individual tissues interact or influence the altered hormonal, physiological, metabolic and immunological rearrangements in biological systems toward manifestation of specific diseases are among major knowledge gaps that require systematic studies.

**Fig. 1:**
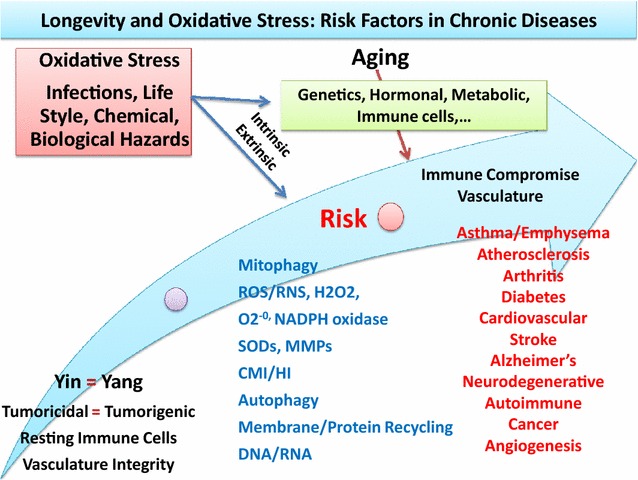
Schematic representation of maintenance of Yin vs. Yang (tumoricidal vs. tumorigenic) of acute inflammation in health (*left panel*, Yin = Yang) or the induction of oxidative stress in alterations of intrinsic and extrinsic factors [e.g., loss of mitochondrial function (mitophagy) or ribosomal function (autophagy), accumulation of oxidants (ROS/RNS), mutated genes (DNA/RNA) immune suppression (changes in CMI/HI)] toward increased risk of chronic diseases (e.g., asthma/emphysema, diabetes and cardiovascular diseases or cancer and angiogenesis) during aging process (*right panel*); see text

In general, the age-associated senescence in organ systems and initiation or progression of a wide range of chronic diseases involves minor or major adaptations or alterations in the integrity of architecture (morphology), function (behavior), biochemical, bioenergetics, immunological (cell mediated or humoral or CMI-HI), mechanical and physical properties in tissues/cells that alter the intra-, extra-cellular components including declines in oxygen consumption [4–9, 36–60]. Among age-associated biological readjustments in tissues/organs, the sustained oxidative stress (sub-clinical, unresolved inflammation) perhaps has the most adverse cumulative influence in immune and non-immune cells (e.g., APCs, T and B cells, epithelium, endothelium, GCs) responses that would damage cellular components (e.g., membrane, cytoplasm, chromosome, mitochondria, ER, lysosome, Golgi apparatus) and related molecules (e.g., DNA/RNA, epigenetic modifications, enzymes, proteins, cytokines, lipids) in vertebrates, invertebrates and humans [4–9, 61–80]. Examples are the reported data that strongly suggest that continuous (chronic) up-regulation of pro-inflammatory mediators (e.g., TNF-α, IL1β, COX-2, iNOS) are due to redox imbalance that activates anti- inflammatory signals including NF-kB, IL-10, PGE2, IL-5 in an attempts to terminate inflammatory conditions. However, the mismatch expression and co-expression of Yin and Yang mediators in the host tissue result in initiation of chronic diseases [4–8]. A detailed analyses of data on age-related chronic diseases reveals that oxidative damage has profound influence over the bioenergetics of tissues and the function of mitochondrial metabolism (see below) (Fig. 1) [6, 80–90].

In discussing biology of age-associated chronic diseases it is helpful to keep in mind the hypothesis that chronic inflammation differentially influences the tissues that are immune-responsive (e.g., epithelium, endothelium, vasculature, mucus secreting goblet cells, stroma) or immune-privileged (e.g., BBB, CNS, neuroretina, uterus, testis) in initiating multistep disease processes such as site-specific cancers or neurodegenerative or autoimmune diseases [8].

It is also important to note that from birth toward adulthood and aging or during induction of diseases, the multi-cellular organ systems of the human body is subject to continuous energy-demanding wear and tear processes in order to accommodate the Yin and Yang events and the repairing, remodeling and adaptation of tissues. Aging process itself is a dynamic phenomenon of biological regeneration and degeneration characteristic of all multi-cellular organisms, presenting minor or major declines or skewed/retarded or accelerated biological switches [4, 6, 13–23, 33–40, 51, 54–56]. In general, the age-induced altered natural biological and immunological activities would lead to altered effectiveness of immune surveillance, the balance between tumoricidal (Yin) vs. tumorigenic (Yang) properties of immune system, weakening the body’s ability to respond to new stimuli that potentially threaten the body’s survival. However, further analyses of data suggest that the ancestral/innate compartment of immune system (e.g., MCs, NKs, DCs, MΦs) is relatively preserved in older individuals compared with the more acquired sophisticated and complicated adaptive immune cells (e.g., T and B cells, Treg, Th1/Th2, CD4+/CD8+) that undergo profound modifications throughout life [4, 6, 11, 13–15, 23, 58–61, 66–72].

## Bioenergetics and biological rhythms in health, aging process and diseases

**Fig. 2:**
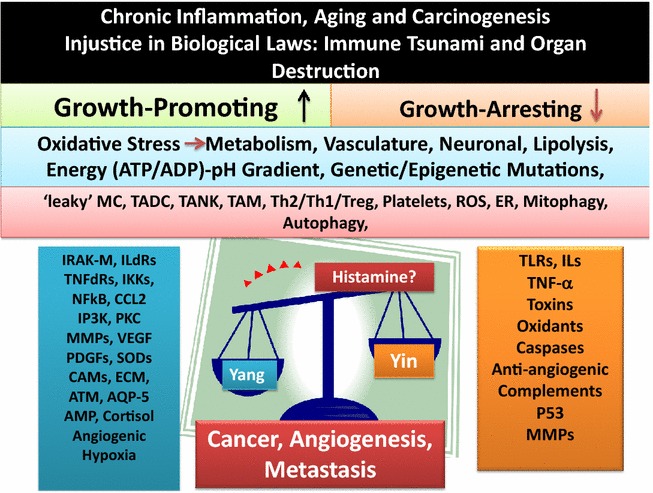
Schematic representation of the role of chronic inflammation in aging and induction of immune tsunami in multistep carcinogenesis. The overall scheme shows that loss of balance in tumoricidal (Yin) and tumorigenic (Yang) properties of protective acute inflammation (effective immunity) is associated with mismatched exaggerated expression and co-expression of apoptotic and wound healing factors. Circulating histamine release at low levels is hypothesized to play important roles in the induction of tumorigenesis and angiogenesis (see text)

A brief overview on extracted information on the complex and well orchestrated bioenergetics that are required for maintenance of biological activities of living systems is helpful to propose a working model for age-associated diseases and carcinogenesis. The functional importance of mitochondria, the double membrane-bound organelles in most eukaryotic cells, in diverse energy-dependence of immune-biological, physical and mechanical dynamics of organ systems in health and age-associated illnesses are outlined below ([4–8, 32, 36, 40, 53, 71, 74–100], manuscript in preparation) (Figs. 1, 2):

1. The body is a dynamic system of complex and highly interactive multiple organs, whose function continually evolves, from the conception and fetal growth and development all the way through life including the aging and disease processes. Aging and weakened status of controlled wear and tear processes (regeneration, degeneration and recycling events) are likely the bases for initiation and progression of diseases and death.
2. The proper functioning of organ systems involves a wide range of well-orchestrated and energy-dependent physical, mechanical, molecular and chemical signal transduction mechanisms involving genetic/epigenetic, enzymatic, metabolic and neuronal activities for the purpose of regeneration, degradation and turnover processes and survival. The signal transduction mechanisms are defined as the highly regulated cross talks or communications between and among organs, glands, membranes and organelles of tissues/cells such as the skin, liver, kidney, lung, heart, stomach, thymus, and the innate and adaptive immune cells, vasculature, neuronal, and gastrointestinal tract.
3. Among critical steps in sustenance of normal living cells is the proton pumping across the membrane and generation of differential acidity among extra-, and intra-cellular membrane components and cytoplasm. Continued proton pumping and the generated electricity is required for numerous routine cellular activities such as stimuli-induced expression of danger signals and activation of immune cells, degradation and growth-arrest of defective cells (e.g., cancerous cells), repair of inappropriate protein and mutated DNA/RNA synthesis, detection and destruction of pathogen’s structural components for immune cell recognition and activation, cellular proliferation, wound healing and growth, as well as ion/solute transports, lysosomal digestion and protein recyclings and the mitochondrial function. For example, the extent of proton pumping provided through vascular or cellular membrane by ATPases alters to varying degrees, from the time of fetal growth and after birth and during aging or disease processes including carcinogenesis. Furthermore, agents that are considered foreign elements and the aging process can temporarily or permanently disturb the membrane effective potential of cell environmental pH levels, causing skewed signal transduction and crosstalk between and among cells/tissues for achieving and controlling the required activities of the cells.
4. Interactions and signal transductions between immune and non-immune tissue components (e.g., cells, organelles, proteins/enzymes, lipids, neurons, DNA/RNA) are amazingly successful complexes of turnover and regeneration processes with durations lasting from a fraction of seconds to several minutes; few days or months and even years. To achieve various cross talks, all types of cells and organ systems must follow biological rules of rhythmicity at different and precise levels. The links among physio-immune-neuro-pathologic features of nearly all living species follow the control mechanisms of circadian rhythms (positive–negative response cycles) or biological clock and ‘biological laws’. The circadian system is defined as a principal pacemaker in the suprachiasmatic nucleus (SNC) in coordination with a number of peripheral circadian oscillators. Patho-physiological features of chronic diseases such as metabolic syndromes, diabetes and cardiovascular complications, neurodegenerative and autoimmune diseases or site-specific cancers are very likely associated with different degrees of disruption in the ‘biological laws’ that control certain components of the circadian cycles. The insufficient circadian rhythms could be the results of mutations or deficiencies of the clock genes or circadian control genes that consequently influence physiological, neurological and immunological behaviors in organ systems; altering synchronization between SCN and other peripheral dual features of biological oscillators.
5. The various synchronization properties of biological systems are defined as the natural positive and negative duality of (oscillations) or biphasic events that are required for maintenance of health. In general, approximate duration of biological rhythms (collectively labeled as ‘effective acquisition time’) are classified into three major categories:
  - Ultradian, lasting seconds to several hours (e.g., neuronal and visual signal transduction, ion fluxes, hormonal release, enzymatic reactions, transporter activities, biosynthesis of receptor molecules, chromosomal repair activities, type 1, immediate hypersensitivity responses).
  - Circadian, lasting approximately 24 h (e.g., skin turnover, melatonin, cellular and extracellular membrane component biosynthesis),
  - Infradian, also known as tidal or annual pathways, lasting more than a few days (e.g., biosynthesis and turnover of hemoglobin, mast cell sensitization and effector responses). Regeneration of complex organ systems (e.g., liver, lung or kidneys) may take a few years to be completed.

As detailed below, the author’s definitions of Yin (tumoricidal) and Yang (tumorigenic) properties of effective immunity may serve larger applications for understanding of the biphasic (dual) activities of system biology in sustenance of health or initiation of diseases [4–8].

## Is cancer a severe accumulated delayed hypersensitivity reaction of immune-responsive tissues? A daring hypothesis or common sense rational for finding answer to cancer?

Reviewing a large body of evidence on many chronic inflammatory diseases and carcinogenesis the author proposes that cancer is a severe form of hypersensitivity responses (immune disorder) in site-specific tissues resulting from accumulation of exaggerated expression and co-expression of immune responses and creation of a molecular immune tsunami, primarily in the immune-responsive tissues. However, as we recently suggested [4, 8] oxidative stress-induced loss of architectural integrities and function of immune-privileged tissues (e.g., BBB, neuroretina, cornea, CNS, uterus, testis) could shift these immune-protected (ignorance) tissues to induction of local immune-responsiveness in the direction of neurodegenerative diseases or multistep carcinogenesis. Figure 2 depicts that longevity and chronic or unresolved inflammation (oxidative stress) cause loss of balance in natural protective mechanisms of effective immunity provided through Yin (tumoricidal) and Yang (tumorigenic) pleiotropic properties of acute inflammation. The scheme represents that oxidative stress and exhausted-‘leaky’ mast cells (MCs) could induce release of low level histamine (independent of antigen-specific IgE-FcεR binding and MCs degranulation) in the host environment (or circulating blood), leading to activation and infiltration of other immune cells [e.g., eosinophils, tumor-associated macrophages (M2 phenotype or TAMs), tumor-associated dendritic cell (TADCs), tumor-associated natural killer cells (TANKs), or activated T (Treg, CD4+/CD8+) and B/plasma cells] in an attempt to terminate the inflammatory conditions, which normally occur during Yang (tumorigenesis) events of self-terminating acute inflammation [4–9].

As noted above, the overall control switches of the stimulating and inhibitory processes that follow the biological rhythmic rules are perhaps larger applications of the original definitions that the author proposed for effective immunity. Earlier we defined that acute inflammation possesses 2 highly regulated and biologically opposing arms (biphasic or Yin and Yang) properties of effective immunity (immune surveillance) for protecting the body against all biological, chemical and environmental agents that are perceived harmful (foreign agents) to the body’s maintenance of health throughout life [4–8].

Recently, re-analyses of our original data on experimental models of acute and chronic ocular inflammatory diseases that were established in 1980s, the author reported the first evidence [5] on early sequential interactions and synergies between host/resident and recruiting immune and non-immune cells (e.g., MCs, GCs, B cells, TAMs, epithelial and vasculature) in conjunctival-associated lymphoid tissues (CALTs) that resulted in cell growth, tumorigenesis and angiogenesis [5, 6, 72, 100–108]. The results demonstrated the first evidence on antigen-(immune disruptor-) induced earliest triggering events that included the release of histamine and prostaglandins (e.g., PGF-1α, stable form of PGI2) during the time course kinetics of identifiable phases of immune dynamic alterations toward multistep tumorigenesis and angiogenesis, [4–8, 100]. It should be noted that despite numerous publications that are available on the role of low level carcinogens (immune disruptors), chemical, environmental and biological agents that influence induction of site-specific cancers, no other reports demonstrated the earliest events that occur in changes of immune response dynamics in host tissue that would lead to multistep carcinogenesis [6, 109]. In our studies the contribution of histamine receptors, biosynthesis of IgE and other immunoglobulins and IgG isotypes (e.g., IgG1/IgG2), activation of B/plasma lymphocytes, infiltration of eosinophils and macrophages, and the involvement of vasculature at various stages of inflammatory responses such as edema during acute, intermediate (down-regulation phenomenon) and chronic phases of inflammatory responses toward multistep tumorigenesis and angiogenesis were demonstrated. We also demonstrated that the eyes of newborn animals from highly sensitized parents were reactive to the first or second challenges toward the antigen [6, 100], suggesting maternal transfer of immunity and possible altered genomic stability of the fetus. Confirmation and extension of these fundamental preliminary observations should provide key information on the role of circulating histamine in potentially altering the resting status of immune and non-immune cell activities including genomic stability of the organ systems after birth and in adulthood or aging processes.

We proposed that continued presence of oxidative stress (e.g., repeated stimulation of conjunctiva) causes expression of mismatch apoptotic and growth promoting factors that would create an immunological chaos or immune tsunami in host tissue and damage the architectural integrities and functions of tissue components [e.g., epithelial thickening and/or thinning (growth and necrosis), epithelial-mesenchymal transition, loss of lymphoid tissue capsular boundary, exhausted or ‘leaky’ MCs (TAMCs), eosinophils infiltration, TAMs, activation of GCs] in CALTs during developmental stages of tumorigenesis and angiogenesis [4–8, 100, 101]. It was also suggested that oxidative stress in other host tissues, such as gastrointestinal track (gut-associated lymphoid tissues-GALTs) or lung airways (lung-associated lymphoid tissue-LALTs) whose host defense mechanisms (e.g., epithelium, MCs, B cells, mucus secreting GCs) are somewhat comparable to CALTs, would induce expression of similar mismatch immune responses leading to induction of early and late phase immune (hypersensitivity) responses causing accumulation of site-specific tissue injuries in the direction of cell growth promotion, pre-neoplasia, polyps or cancer and angiogenesis. In such tissues, it is very likely that the early events in triggering alterations of immune responses are the release of histamine, biosynthesis of prostaglandins or leukotrienes and perhaps other acute inflammatory mediators and respective receptor molecules that also contribute to the activation and expression of vasculature components (Fig. 2) [4–8]. The above mechanisms may also be extended to tissues whose primary cell compositions are not necessarily mast cells. In this context, it is possible that continued activation of other immune cells (e.g., MΦs, DCs or B and T cells) provide signals for activation of MCs at peripheral tissues for the release of low level (circulating) histamine into host tissues in an attempt to terminate inflammatory conditions causing further damages to the target tissue.

In summary, maintenance of health depends on the inherently precise expression and regulations of energy-requiring positive and negative biological control switches (biological rhythms or clocks) between the local (host) and distant immune and non-immune systems such as vasculature, metabolic, neuroendocrine or neuro-physiological pathways.

Rational for the hypotheses that cancer is a severe form of accumulated delayed hypersensitivity reactions and the roles that circulating low level histamine play in sustaining oxidative stress toward tissue growth promotion comes from the analyses of reported data on basic and clinical studies showing direct or indirect contribution of histamine in a wide range of age-associated immune disorders or tumorigenesis and angiogenesis as outlined below [4–8, 100–108, 110–148].

## Role of mast cells and histamine in increased allergies during aging, immune disorders and carcinogenesis

In this section, the role of activation of MCs, as effector cells (with pharmacological responses) within the innate immune system is briefly reviewed to propose that the histamine release, at different inflammatory conditions such as acute, intermediate and delayed hypersensitivity reactions contribute to varied expression of ‘mild’, ‘moderate’ or ‘severe’ immune disorders, including the induction of tissue growth and tumorigenesis and angiogenesis. Details on the role of other innate or adaptive immune cells (e.g., NKs, DCs, MΦs, basophils, T and B cells) in the induction of inflammatory diseases or site-specific cancers are beyond the scope of this article.

It is well documented that older adults often demonstrate increased and diverse allergies such as asthma, emphysema, COPD, ocular allergies (e.g., vernal conjunctivitis), skin allergies or atopic dermatitis [4–8, 100–108, 110]. MCs are primarily recognized as the key cells in immediate or type 1 hypersensitivity reactions and/or delayed type immune disorders. The distribution of MCs throughout serosal and mucosal tissues and their close proximity to blood vessels suggest their involvement in pathological conditions of several diseases involving increased angiogenic activity. A role for MCs has been documented in many allergies or other mild or severe immune disorders such as psoriasis, atherosclerosis, rheumatoid arthritis, gastritis, as well as haemangioma, neoplasms and tumorigenesis [4–8, 100, 110–148]. It is possible that the initiation of other neurodegenerative and autoimmune diseases is partly due to the presence of low level (sub-clinical) circulating histamine. We suggested that the rise in number of MCs in several oxidative conditions, was due to oxidative-stress-induced production of unscheduled (not fully granulated, immature MCs) or the increased number of degranulated or exhausted (‘leaky’) mast cells causing release of low level histamine independent of antigen-specific IgE-fcεR aggregation of activated MCs that would signal for the activation of eosinophils, TAMs leading to multistep angiogenesis and tumorigenesis [4–9, 100, 101].

For a century, histamine has been shown to cause smooth and cardiac muscle contraction or bronchoconstriction, and symptoms of anaphylactic shock that required pharmaceutical industries to develop clinical antihistamine drugs. Histamine receptors (H1-H4) are involved in the activities of vasculature, muscle constriction and neuronal tissues and contribute to a wide range of effects in neuronal tissues and immune and non-immune cells or metabolic profiles as well as pathophysiology of autoimmune and neurodegenerative diseases and tumorigenesis [4–6, 100, 110, 133, 135]. Stimuli- (immune disruptors)-induced acute inflammatory responses release pre-formed or newly synthesized mediators, from effector cells (e.g., MCs) to rapidly cause mucosal edema and mucus secretion, constriction of smooth muscle and/or hyperpermeability responses and activation of vasculature to facilitate other inflammatory cell infiltration for wound healing events or production of gastric acid in gastrointestinal tract for proper digestion of foods. Histamine (catecholamine), an alkaline and a vasoactive agent is synthesized through the actions of amino acid histidine decarboxylase, (l-histidine decarboxylase-HDC) that is present throughout the body. There are at least four types of histamine receptors that produce varying physiological effects such as vasodilatation, gastric acid secretion, brain/neuronal activation and cardiovascular actions [6, 110, 136–148]. For example, in the stomach, histamine is stored in hormone-producing cells such as enterochromaffin-like (ECL) cells and can be released through ECL chemokines receptors (CCK2) to activate H2R and initiate signals for somatostatin receptors to secrete acids (e.g., pepsin, HCl). The gastric acid secretion is influenced by complex signals from endocrine, paracrine and neurocrine primarily through gastrin-histamine, CCK-somatostatin and acetylcholine. Therefore, histamine plays important roles in the control of acid secretion, mucosal integrity, gastric function and maintenance of health [110, 142].

Recently, Cundell and Mickle [110] presented a comprehensive review on the roles that histamine and its receptors play in the induction of a wide range of immune disorders. They also presented the degrees of effectiveness of many antihistamine agents in the last several decades. The search for more effective remedies continues to date. The effective pharmacological levels of histamine to cause pro-inflammatory effects in ocular tissues, skin, gastrointestinal or lung respiratory or neuronal tissues are much greater than its normal circulating level (<0.3–1 ng/ml), ranging from 3 to 12 ng/ml and reach as high as μM levels during stimulation of mucus secretion [6, 110].

Sifting through a wide range of reported data, examples of the diverse effects of histamine and its receptor molecules on metabolism and function of tissues and its roles in immune disorders, cell growth promotion or cancer and angiogenesis are outlined below (Fig. 2) [4–8, 100, 101, 110–148]:

- *Chemotactic Factors (CCXs)*: Histamine release influences neutrophils and eosinophils through expression of neutrophil chemotacttic factor (NCXF) and eosinophil chemotactic factor-A (ECXF-A), perhaps primarily due to activation and degranulation of MCs. We demonstrated that continued MCs degranulation resulted in exhaustion of MCs (‘leaky’ MCs) followed by heavy eosinophils infiltration into the epithelial tissues and mucus-secreting goblet cells, as well as infiltration of TAMs in the conjunctival-associated lymphoid tissues (CALTs) that led to multistep tissue damage, cell growth and tumorigenesis and angiogenesis. Continued activation of MCs could also induce factors such as IL5, and oxidants creating an immunological chaos and recruitment of TAMs during induction of Th2 immune profiles and immune suppression and progressive damage in target tissue.
- *Heparin*: Heparin (mucopolysaccharide) is an acidic proteoglycan and a major component of MCs for neutralizing the alkaline mediators such as histamine and serotonin within the MCs granules. Upon MCs degranulation, heparin is released to inhibit blood coagulation. Analyses of relevant data suggest that protamine, an inhibitor of heparin-dependent activation of proteases (β-tryptase) from MCs also contribute to the resolution of inflammation. Histamine-heparin actions on platelets aggregation during vascular permeability could be quite important, particularly with regard to cancer drugs-induced venous thromboembolism and related complications that are often life-threatening and require additional treatments.
- *Platelet activating factor of anaphylaxis (PAF-A)*: PAF-A is the most potent bronchoconstriction and vasodilatation factor capable of inducing shock symptoms as well as activating platelets to also release histamine and serotonin during hypersensitivity reactions. The action of PAF that originates from vasculature is likely to enhance histamine actions.
- *Bradykinin*: Bradykinin is a vascular nanopeptide and somewhat a weaker alkaline compared with histamine and acts similar to histamine, perhaps contributing to the drop in the blood pressure during acute inflammation.
- *Serotonin*: Histamine causes the release of serotonin another alkaline component from MCs to facilitate smooth muscle contraction and increase vascular permeability in rodents.
- *Histamine effect on de novo inflammatory mediators*:
  1. *Prostaglandins* (PGF1-α or PGI2) are synthesized at very low levels as the consequence of MCs activation and membrane arachidonic acid (AA) metabolism via activation of cyclooxygenase pathways. Their actions are similar and perhaps complementary to the action of histamine in promoting bronchoconstriction in the lung, for recruiting other inflammatory cells such as neutrophils, eosinophils, basophils and monocytes to the site of injury. At least 2 types of PGs, PGF-1α (stable form of PGI2) and PGE2 are involved in Yin (tumoricidal, initiation) and Yang (tumorigenic, termination) events of acute inflammatory conditions, respectively. Induction of PGE2 as contributing factor in the induction of immune suppression and carcinogenesis has also been reported.
  2. *Leukotrienes* (LTs, 1–4) are also synthesized at very low levels from activated MCs membrane AAs metabolism and activation of lipooxygenase pathways. Like PGs, the actions of LTs are somewhat similar to histamine and facilitate vasoconstriction, prolonged smooth muscle constriction and chemotaxis of inflammatory cells such as eosinophils.
  3. *MCs proteases* (e.g., tryptase, β-tryptase or MCP-6, chymase) seem to contribute to both Yin and Yang arms of inflammation. They participate in digestion and clearance of parasitic infections (e.g., Ascaris Suum) and bacteria (e.g., *Trichinella spiralis* or *Klebsiella pneumonia*), as well as digestion/degradation of IgE, to potentially facilitate termination and resolution of inflammation. Whether these proteases also contribute to degradation of histamine receptors at the termination phase of an acute inflammation is not clear.
  4. *Cytokines* A wide range of mediators are expressed and secreted as intercellular signals from activated MCs or other immune and non-immune cells during Yin and Yang processes. They function as tumoricidal (apoptotic) or tumorigenic (wound healing) agents to initiate and terminate and resolve an inflammatory condition. Production of cytokines and chemokines during MCs activation, under various experimental conditions, include pro-inflammatory mediators or danger signaling molecules such as toll-like receptors TLRs (TLR-4), histamine, interleukins (ILs), tumor necrosis factor and receptor molecules (TNFR), oxidants (e.g., ROS, H_2_O_2_, O^2−^) and oxidases (e.g., caspases, NADH oxidase), as well as, post-inflammatory mediators (e.g., IL-5, IL-10, VEGF, FGF, MMPs, FoxP3+) and signals for interactions with T regulatory (Treg) surface proteins (CD4+ CD25+).
  5. *Adenosine* Histamine receptors (H1 and H2) play crucial roles in cardiac muscle function. Adenosine and its agonists (e.g., N6-cyclopentyladenosine-CPA) were shown to reduce the chronotropic and inotropic effects of histamine in human ventricular papillary muscle. The role of histamine-adenosine release and interaction through the expression of H1 or H2 in heart ischemia is not fully understood. Whether histamine potentially contributes to the acid–base balance and the induction of heart muscle contractile forces as the consequence of adenosine-induced low energy, reducing cAMP accumulation and ischemic heart, and/or interactions with norepinephrine and Ca^+2^ flux are important knowledge gaps that deserve further studies.
  6. *Calcium* Ca^+2^ fluxes in energy-dependent lysosomal protein recycling pathways play important roles in protein-membrane recycling events. Release of histamine contributes to the activation and exocytosis of lysosomes and influence Ca^+2^ fluxes from the stored-operated Ca^+2^ channels during MCs activation. Whether chronic stimulation of MCs or MΦs and the release of low level histamine influence the dynamics of free vs. stored Ca^+2^ in cells during induction of ‘leaky’ MCs (TAMCs) or M2 (TAMs) phenotype and the multistep carcinogenesis are also important knowledge gaps that await future studies. Another provocative but logical question is to what extent oxidative stress-induced impaired activities of Ca^+2^ or other ionic channels would influence mitochondrial oxidative phosphorylation and metabolism in aging? (see below, manuscript in preparation).

We suggested that the initial ocular clinical responses (strong or weak) and the nature/potency and extent of activation of MCs (frequency of exposure to antigen) and release of histamine and prostaglandins in conjunctival-associated lymphoid tissues (CALTs) could determine the status of resolution of inflammation (acute hypersensitivity responses) or the induction of multistep tumorigenesis [4–8, 100–108].

The interesting findings on the elevated plasma activities of histaminase (diaminase or diamine oxidase), L-Dopa decarboxylase or calcitonin, in small-cell carcinoma of the lung, or C-cell hyperplasia of familial medullary thyroid carcinoma that were suggestive of embryonic origins that ‘small-cell lung carcinoma and medullary thyroid carcinoma, and further associate histaminases with neuronal crest tumors’ [122–125] are potentially important clues to better understand the origin and susceptibility of organs toward carcinogenesis. Potential alternative or complementary mechanisms are the possibility that plasma histaminases activities are responses toward clearance (removal) of circulating or tissue histamine.

In brief, among many physiological roles that histamine play in tissues, are its direct or indirect dual functions in influencing the vasculature activities, mucosal integrity and function and acid–base balance and the induction of a wide range of immune/inflammatory conditions (immune disorders) including carcinogenesis and angiogenesis [6, 100, 110, 137–142].

## Vasculature synthesis and function in fetal (embryonic) growth and development, allergies, immune disorders, aging and cancer

Vasculogenesis or high endothelial venules (HEV) is the earliest events during the fetus growth and development. In early fetal development, the formation of blood vessels are needed for lymphocyte trafficking and development of secondary lymphoid organs in gut’s Peyer’s patches (also perhaps in lung and conjunctiva or other lymphoid tissues) and for delivery and exchange of nutrients and oxygen to the growing fetus [149–154]. In the process of fetal development HEV are key players in recruiting naïve and memory lymphocytes to lymph nodes (LN) from the blood circulation. There are reports suggesting an enhanced maternal blood supply to the uterine vessels, at the later stage of pregnancy that compete for shared maternal cardiac output toward fetal-placenta and other organs of the mother [148–150]. In addition, new vessel development, neovascularization and angiogenesis are characteristics of vasculature function in normal physiological events for growth and development and during acute or chronic allergies or other immune or metabolic disorders such as neurodegenerative and autoimmune diseases, diabetes and cardiovascular complications, stroke, allograft, infections, as well as carcinogenesis [4–8, 111–114, 140–145, 154–170].

With regard to inflammation, it is suggested that the initial triggers for activation of vasculature are expression of histamine receptors. Several studies on acute inflammatory responses involving MCs degranulation and diverse histamine roles directly or indirectly support this notion [4–8, 59–61, 64, 72, 100–106, 110–114, 137–147]. However, as discussed above, direct evidence on the duality of histamine function in time course kinetics of developmental stages of carcinogenesis and angiogenesis are limited due to the lack of systematic investigations [4–8, 100]. It is well documented that activation of vasculature is necessary for increased vascular permeability (hyperpermeability) and infiltration of inflammatory cells to the target tissue during acute inflammation. As also outlined above, immune disruptors would induce activation of vasculature and host tissue to express a wide range of mediators and respective receptor molecules, such as heparin and histamine receptors, TNFRs, ILs, IFNs, arachidonic acid pathways and synthesis of PGs (e.g., PGF1α, or PGE2) or LTs, cell adhesion molecules (CAMs), vascular endothelial growth factor (VEGF), endostatin, epidermal growth factor (EGF), membrane metaloproteases-MMPs, during Yin (tumoricidal) and Yang (tumorigenic) activities of inflammatory response processes [4–8, 100]. Other events such as activation of histaminases (diamine oxidase), heparinase or inhibitors of prostaglandin synthesis or NOS also contribute to the vascular activation and inflammatory processes. These events are required for regulation of vascular permeability and passage of blood components for their actions (e.g., proliferation, differentiation and infiltration of inflammatory cells, plasma proteins, biosynthesis of complement cascades, platelets activation) at the site of injury during apoptosis and wound healing processes and termination/resolution of acute inflammation. Activation of vasculature (e.g., hyperpermeability reactions, neovascularization and angiogenesis) also participates in a wide range of age-associated allergies and immune or metabolic disorders such as diabetes and cardiovascular complications atherosclerosis, arthritis, neurodegenerative and autoimmune diseases, as well as carcinogenesis [4–8, 67–70, 100–106, 110–115, 129, 154–170].

Therefore, considering vasculature as the tree of life, in general, vasculature has crucial roles in maintenance of health from fetus growth and after birth for development to adulthood, all the way to the aging and disease processes. The fundamental function of vasculature may be outlined in three basic categories:

- Delivery of nutrients and oxygen to tissues/organ systems and removal of gases and waste products from the tissues from the early fetus growth, after birth and throughout life;
- Gatekeeper of immune cells proliferation, differentiation and infiltration of inflammatory cells into stimulated (infected or injured) targeted tissues, facilitating both apoptosis (Yin) and wound healing (Yang) processes;
- Maintenance of crosstalk between neuronal-immune-metabolism and organs/tissues;

## Bioenergetic requirements at different stages of life: orderly growth and development of fetus, healthy adult, aging process and disorderly growth of cancer

It seems that the laws of thermodynamics of open systems apply to the biology of body’s growth patterns from the fetus growth and development to the adulthood and aging process as well as the diseases including cancer. Here, with regard to the role of immune surveillance and inflammation a brief comparison between energy requirements of fetal tissue growth and development will be made with the bioenergetics in healthy adulthood, the aging process and multistep carcinogenesis. An overview of the analyses of a large body of relevant information suggests that the lawless-disorderly growth of cancer cells and angiogenesis, peculiarly share special features with the orderly fetal growth development, vasculogenesis and organogenesis. This comparison will be useful in proposing and presenting a working model for future studies of cancer and the important role that effective immunity play in control of cancer growth. The followings are highlights of comparison in the growth features of fetus and those of cancer cells. Details of data that directly or indirectly support the framework for the present hypotheses are found in examples of the reviews cited in this perspective [4–8, 110–116, 147–190].

1. During normal fetal growth there is an increased expression of several growth promoting events for orderly growth and development of organs. These growth promoting events require minimum oxygen tension supplied through maternal circulation and exchanged through placenta. The fetus growth condition resembles the hypoxic conditions and angiogenesis that cancer cells utilize for their enhanced lawless growth requirements.
2. The reduced oxygen tension (hypoxic condition) during both the fetus growth and carcinogenesis suggests that mitochondrial function is not required (not developed) during orderly fetus growth; and it is dysfunctional in carcinogenesis. Mitochondrial dysfunction (mitophagy) and impaired oxidative phosphorylation are features of nearly all site-specific cancers.
3. A special feature in fetal tissue that, at first appears different from cancerous cells is the role of immune cells. The fetus tissues are naïve with regard to immune cell development. In the growing fetus, naïve or undifferentiated immune T and B cells are transported from placenta through fetus circulation to develop the fetus lymphoid organs. Fetus, being fed in the protective environment of placenta, is not directly exposed to the atmospheric oxygen, and the growing mass is not challenged by the environmental hazards. Therefore, there is no need for an effective immune surveillance (Yin and Yang). Peculiarly, loss of immunity and chronic inflammation similarly causes cancerous cells to escape immune surveillance for their lawless growth. Cancer cells hijack the natural pleiotropic properties of immunity (exaggerated expression of growth promoting factors and selective expression of apoptotic factors) to satisfy their enhanced growth requirements and invasiveness during metastasis. Therefore, absence of effective immunity provides a similar growth condition for promotion of growth of the cancerous cells, a situation that is comparable to orderly growth of fetus.
4. Under the condition of fetus growth, energy requirements for the orderly growth of fetus organs are satisfied by low energy (ATP) production from glycolysis, a somewhat comparable growth feature that applies to cancerous cells and the Warburg effect or enhanced glycolytic pathways for energy production from ATP.

Therefore, it seems that in general the growth and energy requirements for the orderly fetus growth and the lawless growth of cancerous cells are peculiarly comparable. The energy demands for growth of both fetus and cancerous tissues are supplied by low energy requirements from the Yang (tumorigenic) arm of acute inflammation, also a characteristic for wound healing and termination events in normal adults. In the environmentally protected space of fetus growth, there is little need for high energy demands from the mitochondria required for the Yin (tumoricidal) arm of acute inflammation. Absence of expression of pro-inflammatory mediators and oxidants is perhaps necessary for “one way” growth of fetus tissues because otherwise, as indirectly suggested from clinical and nutritional data, it could result in severe fetal rejection (spontaneous abortion), preterm delivery or other damages to the fetus growth causing alterations in cellular and chromosomal (DNA/RNA mutations or deletion) function during organ development that would lead to premature aging, metabolic diseases or cancers after birth (see below) [149–160, 191–193]. Furthermore, as suggested above the growth features of fetus share the characteristics of lawless cancer cell growth with respect to hypoxic conditions to escape growth-arrest by programmed cell death (Yin–Yang events), otherwise, the growing fetus would be viewed a foreign entity, like any parasite, to be eliminated (see below).

Further analyses of relevant data on pre-, and post-natal development and childhood cancers [e.g., neuroblastoma, B-lineage infant acute lymphoblastic leukemia (ALL), mixed lineage leukemia (MLL), myeloid leukemia-Downe syndrome-ML-DS or medulloblastoma] suggest rapid aging or maturation of prenatal-embryonic tissues, accompanied by increased genomic mutations and progressive genomic instability and fusion, presenting embryonic hyperplastic cell growth patterns that favor abnormal proliferations of cell survival under hypoxic conditions [155–159]. Over expressions of several abnormal/mutated growth factors (e.g., MYC, PI3K, MAPK, erythropoietin receptor-B cell factor-1-EBCR1 or BCR) that are also known to contribute to the multistep carcinogenesis in adult cancers are reported in childhood cancers.

How these embryonic mutations are controlled or remain silent in adults that would later cause site-specific cancers in aging individuals are among important biological knowledge gaps. As noted above, we observed that the newborn guinea pigs from repeatedly sensitized animals demonstrated strong hypersensitivity reactions toward the initial challenges (1st or 2nd) by antigen. However, as we also demonstrated sensitization and activation (pharmacological effects) of MCs during immediate (type 1) hypersensitivity reactions in normal animals occurred after 9–12 days of repeated ocular challenges [4–8, 100]. It is possible that fetus MCs are sensitized through maternal antigen-specific IgE transfer. Alternatively, circulating antigen could be transferred through placenta and cause fetus genetic disposition for allergies.

## Important role of mitochondria in health, age-associated diseases and cancer: beyond energy power sources: a working model hypothesis in support of immune surveillance (Yin and Yang)

Longevity and oxidative stress are associated with generation and accumulation of free radicals such as reactive oxygen species (ROS) and other oxidants. While free radicals are normally present in the mitochondria as part of defense mechanisms during stimuli-induced acute inflammation and they are byproducts of normal metabolism, their accumulation is toxic to mitochondrial function, perhaps signaling to shut down the oxidative status of cells, after participation in the Yin events. Oxidative stress-induced continuous generation and accumulation of ROS could impair mitochondrial function including damaging mitochondrial DNA (mutations) leading to progressive reduction in energy output that are significantly below the required levels of body’s function, including its impaired fighting capacity toward infective agents [4–8]. Furthermore, several reports suggest that oxidative stress inactivates or alters function of critical enzymes [e.g., superoxide dismutases (SODs), catalase, glutathione peroxidase and glutathione reductase] that influence the intrinsic metabolites (e.g., uric acid, bilirubin, SH-proteins, glutathione) or extrinsic reducing agents (e.g., vitamins C, D, E, carotenoids, flavonoids) or other antioxidants, and metal chelating proteins that prevent Fenton and Haber–Weiss chemistry as contributors of age-associated diseases [4–8, 172–191, 194–202]. The reported loss of numerous natural protective mechanisms that are associated with aging and chronic diseases strongly support that the oxidative damage to the tissue components, particularly to the mitochondria play key roles in aging and cancer [172–191]. Furthermore, the reduced output energy from mitochondria is implicated in a number of age-associated health conditions such as loss of memory, hearing, vision, stamina, sarcopenia and tumorigenesis [181–188].

It is therefore logical to hypothesize that the energy requirements and thermodynamics of immune surveillance, or the maintenance of balance between Yin (tumoricidal, high-energy requiring growth-arrest) and Yang (tumorigenic, low- energy requiring growth-promotion) arms of acute inflammation that protect the health of adult body, are vastly different from those needed for orderly growth events in fetus (“one way” growth-promotion) and also the lawless growth of cancerous cells (under hypoxic condition). There are a number of recent articles that discuss cancer thermodynamics and the role of electromagnetic resonance in manipulation of the tumor size [96–98]. However, there is little information to compare the differential high or low energy requirements or thermodynamics of immune surveillance in health or disease processes, particularly carcinogenesis. Furthermore, important biological gaps exist in comparing or understanding the fetus and cancerous cell growth requirements with regard to the molecular dynamics of immune surveillance.

Here we consider that the mitochondrial contributions in the metabolism of tissues and maintenance of health or induction of diseases are far more important than being an energy reserve for the supply of energy. In addition to their roles in TCA cycle and generation of intermediates that are needed for oxidative phosphorylation, there are a number of important metabolic events occurring in mitochondria that include the energy requiring metabolism of essential branched amino acids (e.g., leucine, isoleucine, valine) and biosynthesis of structural proteins. It is hypothesized that the growth of fetus and cancerous cells shares additional characteristics with regard to defective mitochondrial function. Both fetus and cancer cell masses are defective tissues and both tissues may be considered as parasites dependent on their hosts for survival with regard to mitochondria and energy requirements. The growth of both masses, orderly (in fetus) or lawless (in cancer) is dependent on their hosts (maternal-placenta, or site-specific tissues), due to the lack of functional mitochondria as proposed below.

It is hypothesized that the primary reasons for lawless growth of cancer masses, and loss of cell–cell contact inhibition are impaired mitochondrial functions including damages to pathways for biosynthesis of proteins containing branched chain amino acids such as alanine, leucine, isoleucine or valine. The proteins that are expressed in cancerous cells, in all likelihood, possess the following interrelated/interdependent defective features:

- Dysfunctional properties due to lack of structural/architectural properties and impaired integrity;
- Impaired ionic or water channel transport properties, that would influence extra-, intra-cellular membrane signal transduction properties (e.g., proteins/lipids recycling pathways, lysosomal hydrolases), as well as cellular anchoring and communication features to neighboring cells, features of contact inhibition;
- Defects in metabolism of branched chain amino acids could additionally damage the balance between oxygen-requiring events in Yin and Yang events of immunity;
- Exaggerated expression of anti-apoptotic mediators that would favor growth promotion and enhanced glycolysis and increased glucose transport and utilization (Warburg effect);
- The above events that are features of cancerous tissues, have shared characteristic with fetus growth as well as wound healing properties of Yang arm of acute inflammation;

It should be emphasized that with an effective immunity (balance in Yin and Yang arms of acute inflammation) the defective cancerous cells are monitored, arrested and neutralized as any other pathogens/parasites or foreign elements (stimuli) that threaten the body’s survival [4–8]. However, aging and oxidative stress could induce inefficient functioning of mitochondria causing impaired metabolism of branched or aromatic amino acids (e.g., phenylalanine, tyrosine) and energy-requiring protein biosynthesis that are needed for proper assembly and architectural integrity of tissues. As noted above, loss of Yin and Yang pathways and exaggerated or mismatched expression of pro-, and anti-apoptotic mediators in immune-responsive tissues could favor growth promotion, enhanced glycolysis, and increased glucose utilization (Warburg effect), events that share features of fetus growth and also wound healing events.

While the importance of individual genetic makeup (intrinsic factors) in longevity and delayed onset of diseases is logical, how the confounding extrinsic factors such as the interactions of oxidative stress and aging process would lead to increased somatic mutations that affect the regulations of biological clocks and functions of organ systems in the direction of diseases are not understood. The degrees of interactions between intrinsic (innate) factors or the genetic makeup that influence the physiological behaviors of the organs/tissues (e.g., vasculature, metabolic, hormonal, neuronal and immune response pathways), combined with extrinsic factors (e.g., exposures to chemical, environmental, biological hazards, carcinogens, life styles) are likely major factors in the aging process that would determine the outcomes of age-associated chronic conditions (Figs. 1, 2).

## Proposed working model to explain immune surveillance: mitochondrial bioenergetics and life cycles

There is now compelling evidence that the normal adult livings is centrally regulated by highly complex biological activities that are energy-requiring for maintaining the organs/tissues wear and tear mechanisms, principally involving sophisticated immune and non-immune system crosstalk including autophagy for protein/lipid or genomic components recycling and turnover processes throughout adult life. When components of effective immunity are defective or become overused by frequent encounters with stimuli, particularly during aging, the organ systems lose, to varying degrees, the precise response dynamics that eventually lead to initiation and progression of a wide range of immune and non-immune disorders including loss of effectiveness toward new challenges or control mechanisms to arrest growth of cancerous cells [4–8, 175–179, 184–190].

**Fig. 3:**
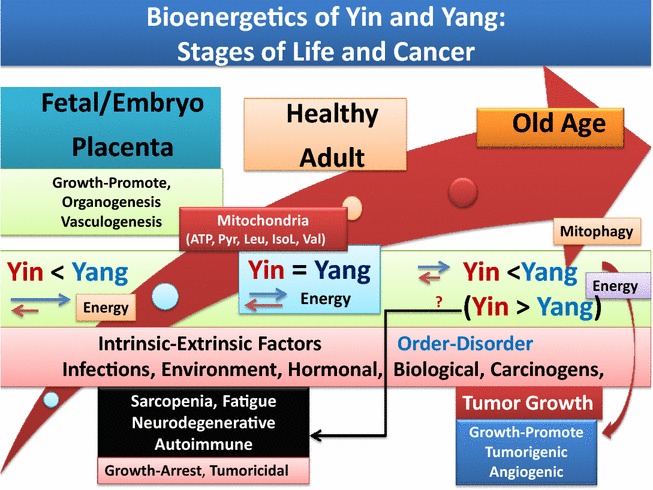
Schematic representation of a working model hypothesis explaining the function of immune surveillance, or Yin (tumoricidal, growth-arrest) and Yang (tumorigenic, growth-promote) of inflammation at different stages of life and age-associated diseases. The role of oxidative-stress-induced mitochondrial damage and the induction of carcinogenesis and neurodegenerative or autoimmune diseases are suggested to lead to differential damages of immune-responsive and immune-privileged tissues (see text)

Digesting reported data support the interrelated series of hypotheses that are presented in this article, with regard to differential roles that mitochondria and immune surveillance (Yin and Yang) play during fetal growth, healthy adulthood and age-associated diseases and carcinogenesis. There are a number of excellent reports on the diverse roles that presence or absence of functional mitochondria play in microbial (parasitic) life or in the mammalian metabolism and function of tissues for survival and maintenance of health, aging process, graft-vs- host immunopathology and other immune disorders or metabolic syndromes and carcinogenesis ([6, 175–179, 184–190, 194–228], manuscript in preparation). Integrating relevant data and connecting the dots on available information, it is logical to propose a working model for the complex biology of immune surveillance and the required bioenergicts at different stages of life, from fetal growth (feeding off its host for survival, like parasites), to post-birth growth and independent growth development to normal adulthood and during aging process and carcinogenesis. The proposed working model presents a rational for future studies on age-associated chronic diseases with emphasis on cancer biology and angiogenesis (Fig. 3).

As depicted from Fig. 3, the conceptual components of the proposed working model briefly constitute the followings:

- With the exception of expression of limited apoptotic factors that are required for digestion of extracellular matrix and related events for organogenesis and vasculogenesis, there is little need for high energy demands of a fully functional mitochondria or immune surveillance during fetus growth and development within the confined protective environment of placenta.
- There are amazingly comparable features with regard to the fetus growth or parasites (e.g., anaerobic flagellate monocercomonoides) and those of cancerous cells, as their growth is dependent on the host with regard to energy requirements and mitochondria. The fetus and anaerobic eukaryotes or cancerous cells similarly utilize inefficient ATP production through glycolysis due to impaired TCA cycle of mitochondria and under hypoxic condition of their growth environment. The exception from mammalian fetus growth and that of parasitic growth comes after birth. The fetus organ systems are capable of becoming independent after birth, perhaps primarily due to establishing a functional mitochondria and development of effective immunity, features that microbials lack.
- The fetus immune systems are collection of naïve T and B cells during organogenesis and the development of lymph nodes, thymus, bone marrow or liver. The developing organ systems of fetus are not subject to external (environmental) challenges that could be harmful to its survival.
- The bioenergetics required for orderly fetal/embryonic growth and organogenesis are primarily supported through limited oxygen supply within the environment of amniotic fluid of placenta. As noted above, under normal fetal growth, a functional immune surveillance is absent and the growth of organ systems utilizes glycolytic pathways for production of ATP (Warburg-like phenomena). The fetus respiratory system is not functional at this stage. Therefore, under normal fetus growth and the low oxygen tension of the placenta, the energy produced and consumed by the growing fetus is sufficient to support the orderly growth of the tissue masses.
- After birth drastic and simultaneous changes occur for adjusting the growth of newborn to the atmospheric pressure and oxygen-dependent and high energy demands that are required for completion of development of organ systems (e.g., lung airways, cardiac muscle, brain, liver, eye, neuronal, lymphatic and vasculature) independent from maternal-placenta. The organ development after birth requires major changes in several biological systems to become physiologically efficient. Perhaps the most fundamental biological changes after birth are completion of mitochondrial development and function for supplying high energy demands from ATP through glucose oxidation and pyruvate-shuttle to the mitochondria, the requirements for establishing biosynthesis of pyruvate carrier proteins/transporters, related enzymes for TCA cycle intermediates during oxidative phosphorylation, as well as metabolism of branched chain amino acids for biosynthesis of structural proteins.
- Mitochondrial pyruvate carriers (MCPs; MPC1, MPC2 or Mpc3, or bakers’ yeast) are the members of mitochondrial inner membrane proteins with fundamental functions. The function of MCPs is fueled by a proton gradient across mitochondria inner membrane. Other fundamental functions of mitochondria include the metabolism and utilization of essential branched chain amino acids (e.g., valine, leucince, isoleucine/norleucine) for protein synthesis that are perhaps crucial for architectural maintenance of cellular integrity and function. Growth defects have been reported in yeast lacking branched amino acids (e.g., leucine and valine).

Analyses of relevant data suggest that the well-defined substructures of mitochondria develop after birth and upon exposure to atmospheric oxygen. In experiments using chick embryos in tissue cultures (under defined oxygen pressure and growth media), we reported that the development of myotubes from muscle myoblasts in the presence of insulin, required alanine or pyruvate and that serine or cortisol (growth promoting agent, hormone) promoted the effects of alanine or pyruvate for development of myotubes as well as expression of creatine phosphokinase activity, a muscle-specific marker for myogenesis [212]. Since myoblasts are highly glycolytic, we suggested that pyruvate was limiting perhaps because aspects of oxidative metabolism was limited in the embryonic cells.

Additional indirect support of the above interdependent hypotheses on the development of functional immune surveillance and mitochondria after birth is that the immunity of newborn baby, at least for the first few months, is provided through the mother [223–226]. The immaturity and immune tolerance in neonatal and the role of stem cells have been the topics of extensive studies for organ transplantation [226, 227]. Except for requirements for breathing and development of organ systems, the infant mitochondria may take a while to be fully functional for performing high energy demands of immune defense properties. Therefore, formation and maintenance of immune surveillance (Yin–Yang) and the required expression of precise quantities of toxins and growth factors that are needed at moment notice when host tissue is challenged, are likely the most energy-consuming processes for normal living, and require fully functional mitochondria.

Among several other energy-driven pathways of biological activities, are the major complex processes involving 2 distinct forms of mammalian target of rapamycin (mTORC1 and mTORC2), an evolutionary conserved family of serine/threonine kinase and member of the family of phosphoinositide-3 kinase (PI3 K) related kinases (PIKK). The role of mTORC1 has been shown to engage with complexes such as NAD+-dependent deacetylase enzymes, SIRT1 pathways and include mediation by ROS-induced activation of S6 kinase and H2O2 production for signal transduction and regulation of cell growth and proliferation involving the immune response crosstalk [4–8, 215, 216, 218, 220]. Abnormal function of TOR contributes to the tissue hypertrophy and hyperactivity toward cellular damage and age-related health conditions. Furthermore, activation of TOR-dependent pathways seems to limit the lifespan by accelerating age-related diseases even before the accumulation of ROS induces death.

The two biologically opposing arms of Yin and Yang that we described for acute inflammation [4–8] are recent concepts. Analyzing data on the roles that antigen presenting cells (APCs) play for sensing danger molecules and expressing appropriate death factors to digest and present processed pathogens to adaptive immunity are dependent on burst of energy from mitochondrial oxidative phosphorylation in Yin (tumoricidal), while wound healing events primarily use glycolysis (Warburg effect) as energy source for termination of inflammation and repair of tissue. The two processes seem to operate with different scale of energy demands (manuscript in preparation). Determination of thermodynamics of the complex specific crosstalk of the immune surveillance provided through Yin and Yang that are essential for promotion and maintenance of health awaits future systematic studies. However, review of reports on thermodynamics of cancer by Lucia and colleagues [96–98] provides important clues into the thermodynamics and electromagnetic features of cancer cells and their potential use for anti-cancer therapy. Considering tissue bioenergetics and the role of immune surveillance, one may conceptualize differential ionic variations are required during Yin and Yang events of acute or chronic inflammation. The charge differences during tumoricidal (Yin) and tumorigenic (Yang) processes include asymmetry in bio-electricity of reactions and differential properties of pH gradients and respective ionic channels (e.g., H^+^/Na^+^ or Cl^−^/H^+^ exchangers), active transport systems or pumps (e.g., Na^+^/K^+^ ATPase, Ca^+2^ ATPase) or water channel (aquaporins) properties, under various inflammatory conditions or carcinogenesis ([4–8, 139–142, 185, 193, 209, 214], manuscript in preparation).

In summary, the overall analyses of relevant data suggest that the mitochondria undergo extensive development after birth for their fundamental functions. It is possible that both the orderly growing fetus masses and the lawless growth of cancer cells are defective tissues (like parasites) with regard to important architectural and structural proteins that contain branched chain amino acids (e.g., Leu, IsoL, val) whose energy-demanding biosyntheses require functional mitochondria. The fetus growth patterns occur with little/no need for mitochondrial oxidative phosphorylation or the high energy-requiring tumoricidal events (Yin). During fetus growth, in the absence of external challenges it is proposed that only one arm (Yang, tumorigenic) of immune surveillance is functional. The features of orderly growth of fetal tissues resemble many of the lawless growth properties of cancer cells when the balance between tumoricidal (Yin) and tumorigenic (Yang) properties of acute inflammation is lost in favor of cancer cell growth. It is likely that longevity and sustained oxidative stress impair mitochondrial function for providing the required energy for maintenance of Yin–Yang balance as well as utilization of the essential branched chain amino acids for proper synthesis, assembly and integrity of structural proteins. Cancer cells, much like the fetus growth (or parasites whose survival are dependent on their host oxidative metabolism), use the inefficient tissue glycolysis for ATP production and supply of energy. The high activity of glycolytic pathways in carcinogenesis is perhaps another reason that cancer cells have increased glucose transport and metabolic activities.

## Answer to cancer and future directions: essential mitochondria for maintaining Yin–Yang of effective immunity, pyruvate shuttle and biosynthesis of proteins and cellular integrity

Special or shared biological complications of full-blown diseases that generally determine the outcomes (manifestations) of nearly all age-associated chronic conditions fall into three major interdependent categories; vascular complications, tissue necrosis or tissue growth. These categories are principally characteristics of defects in Yin (tumoricidal) and Yang (tumorigenic) pathways of acute inflammation [4–8]. Nearly all different biological events that contribute to a wide range of ‘mild’, ‘moderate’ (intermediate) or ‘severe’ allergic reactions, immune disorders or inflammatory diseases also participate in the multistep carcinogenesis.

It is proposed that the histamine release at low (subclinical) levels contribute to the maintenance of oxidative stress and loss of balance in Yin and Yang events and the genesis of immune disorders including carcinogenesis. Whether chronic inflammation-induced continuous presence of low level histamine (an alkali) in circulation alters the ionic dynamics (charge characteristics) and stabilities of extracellular membrane proteins, water channels (aquaporins) or ion transport mechanisms, that also influence the signals within cytosol, intracellular membranes (e.g., ER, ribosome, mitochondria, Golgi) and the bioenergetics of tissues are fundamental topics that remain partially understood. The presence of histamine could influence the acid–base balance or the pH levels of many cellular activities (e.g., gastric or mucus secretion, exocytosis, Ca^+2^ fluxes) or ionic composition of surface proteins that would lead to minor or major alterations in the integrity of intra-, extra-cellular or vascular components and the endothelial barrier function.

It is further hypothesized that impaired biosynthesis of functional proteins involving branched chain amino acids in oxidative stress-induced dysfunctional mitochondria contribute to the enhanced lawless growth of cancerous cells. Fetus orderly anabolic growth and organogenesis seem to share many features of cancer growth with regard to lack/impaired biosynthesis of structural proteins within mitochondria and metabolism of branched chain amino acids under hypoxic conditions. The high-energy consuming pathways for import and metabolism of branched amino acids or puruvate-shuttle mechanisms from cytoplasm to the double-membrane structures of mitochondria, and the crucial TCA biosynthetic events for highly efficient ATP production and oxidative phosphorylation that are required for effective immunity (Yin–Yang), in all likelihood, develop after birth.

Cancerous cells should be considered as the intrinsic cellular defects, whose oncogenic growth features within the organized multilayer of normal tissues are routinely monitored and arrested by the highly efficient properties of acute inflammation (effective immunity) as any other pathogens/parasites, senescent and useless cell complexes that are perceived as ‘foreign elements’ and hazardous materials that threaten the body’s survival. Chronic irritations or sustained injuries to the epithelial or endothelial cells result in exaggerated activation, migration or recruitment of other inflammatory cells to the site of injured tissue that would alter the balance between Yin (tumoricidal) and Yang (tumorigenic) properties of effective immunity (Figs. 1, 2, 3) [4–8].

Highlights of important interrelated biological challenges for future basic and clinical studies toward preventing age-associated diseases, particularly cancer are listed below:

- Older adults are immune compromised to varying degrees. In this context the role of mitochondrial function in supplying the energy for maintenance of effective immunity or balance between Yin (tumoricidal) and Yang (tumorigenic) of immune surveillance is fundamental. Age-associated immune compromises in susceptible tissues would increase allergic conditions (e.g., asthma, emphysema, skin or ocular allergies) or other inflammatory conditions (e.g., colitis, gastritis, hepatitis) as well as multistep carcinogenesis.
- Determining the baseline circulating histamine or histaminases as routine clinical tests should be considered useful markers for the status of health or the oxidative stress and acid–base levels in individuals.
- Whether enhanced lawless growth in cancer cells and associated loss of cell contact inhibition correlates with defective/loss of metabolism of branched chained amino acids (e.g., leucine, isoleucine or valine) and the dysfunction of mitochondria (mitophagy) awaits systematic studies.
- The proper mitochondrial function for their diverse functions including energy consuming pathways for puruvate-transport, TCA intermediate metabolism for supplying high energy demands of programmed cell death and carcinogenesis are fundamental topics for future investigations. The mitochondrial-dependent biosynthetic pathways of protein synthesis and metabolism of essential branched chain amino acids for architectural integrity of cells, anchoring or contact inhibition, in all likelihood occur after birth of individuals.
- Systematic understanding of the initial events that disturb the dynamics of Yin and Yang in the immune-responsive or immune-privileged tissues is crucial for understanding the fundamental biological switches in the oxidative stress-induced loss of balance in growth-arresting or growth-promoting properties of tissue necrosis or growth as well as contributions of activated vasculature components in site-specific tissues.
- The potential role of glucose toxicity and enhanced glycolysis and correlations to mitochondrial altered oxidative damage are fundamental topics for future investigations. Other interrelated studies are the role of stimuli-induced changes in insulin-dependent (e.g., muscle, adipocytes, liver) or insulin-independent (e.g., vasculature, endothelial, neuronal, retinal) tissues for glucose transport and utilization could provide key information not only for the basis of diabetes -related complications, but also the association between glucose toxicity, obesity and diabetes with carcinogenesis.
- Identification of host-pathogen initial interactions and influence on immunity in tissues are important areas of research that require systematic studies.
- Synthesis and consumption of the natural essential branched chained amino acids, and agents such as taurine (small SH-containing peptide) could prove to be beneficial in preventing or delaying age-associated disease processes in general, including reducing the fatigues or sarcopenia, or limiting cancer growth by potentially improving cellular contact inhibition properties, or helping/repairing mitochondrial function. In general, it is suggested that consumption of such agents potentially could correct/lower cancerous cells energy requirements from glycolytic activities under hypoxic conditions.

It is concluded that the majority of age-associated chronic illnesses are very likely preventable or correctable, if the research investment were to focus on understanding of the biology of aging based on the maintenance of effective immunity, balancing the inherent tumoricidal (Yin) vs tumorigenic (Yang) properties of immune surveillance. With regard to care for the elderly in hospital settings, caregiver could significantly improve the care conditions if the infections of the elderly and the need for antibiotics and too many drugs could be reduced by considering and applying some of the principles for promotion of immunity discussed in this article.

## Concluding remarks

*The things you do for yourself are gone when you are gone, but the things you do for others remain as your legacy.* Kalu Ndukwe Kalu.

The fascinating biological events that regulate the body’s multi-organ systems from conception to fetus growth development and adulthood life through aging processes are complex molecular communications between immune and non-immune systems. The highly organized and interdependent network of signals and chemical interactions (crosstalk) among and between organs and tissues constitutes the effective immunity for defending the body against intrinsic or extrinsic components that are perceived hazardous to individual health. Disturbance of such magnificently orchestrated crosstalk in effective immunity by frequent exposures to immune disruptors and aging process could cause retardation and defects in various communications in susceptible tissues causing initiation of ‘mild’, ‘moderate’ or ‘severe’ health conditions, including site-specific cancers that we generally consider here as expression of different levels of immune disorders.

As the population of older adults increase around the globe, the healthcare policy makers and professionals must consider switching from the current culture of sick care that has been very costly to the society and investing on systematic and logical projects involving the complex effective immunity that is responsible for sustaining health.

Detailed studies on confirmation and validation of the many components of the proposed model in this article that the joint development of immune surveillance (Yin–Yang) and mitochondria are required for independent survival of organ systems occur after birth, are fundamental new topics for understanding the dynamics of healthy living, the aging and disease processes, including cancer biology and how to prevent or control it.

Finally, as Shakespeare stated *‘Destiny of human being is determined by love alone’.*

## Abbreviations

APCs: antigen presenting cells
CMI: cell-mediated immunity
HI: humoral immunity
MCs: mast cells
TAMCs: tumor-associated MCs
MΦs: macrophages
TAMs: tumor-associated MΦs
DCs: dendritic cells
TADCs: tumor-associated DCs
NKs: natural killer cells
TANKs: tumor-associated NKs
Treg: T regulatory
PGs: prostaglandins
VEGF: vascular endothelial growth factor
FGF: fibroblast growth factor
CAMs: cell adhesion molecules
AMP: adenosine monophosphate
cAMP: cyclic AMP
BBB: blood brain barrier
CNS: central nervous system
TNF-α: tumor necrosis factor-α
TNFR: TNF receptor
PKC: protein kinase C
TLRs: toll-like receptors
ILs: interleukins
ILdRs: IL decoy receptors
IRAK and IRAK-M: interleukin-1 receptor associated kinase
ER: endoplasmic reticulum
GCs: goblet cells
CALTs: conjunctival-associated lymphoid tissues
COX: cyclooxygenase
AA: arachidonic acid
iNOS: inducible nitric oxide synthase
SODs: super oxide dismutases
ROS: reactive oxygen species
MMPs: membrane metalloproteases
LN: lymph nodes
COPD: chronic obstructive pulmonary disease
PAF: platelet activating factor

## Footnotes

1 Computational biology projects such as NCI/NIH Project, Cancer Bioinformatic Grid (CaBig) that cost millions of dollars produced very little to advance anything beyond putting together an enormous data of what is available on different formats.
